# De novo generation of hit-like molecules from gene expression signatures using artificial intelligence

**DOI:** 10.1038/s41467-019-13807-w

**Published:** 2020-01-03

**Authors:** Oscar Méndez-Lucio, Benoit Baillif, Djork-Arné Clevert, David Rouquié, Joerg Wichard

**Affiliations:** 1 Bayer SAS, Bayer Crop Science, 355 rue Dostoïevski, CS 90153, 06906 Valbonne, Sophia Antipolis Cedex, France; 2 Bloomoon, 13 Avenue Albert Einstein, 69100 Villeurbanne, France; 3 Department of Machine Learning Research, Bayer AG, 13353 Berlin, Germany; 4 Department of Genetic Toxicology, Bayer AG, 13353 Berlin, Germany

**Keywords:** Computational models, Machine learning, Cheminformatics, Gene expression

## Abstract

Finding new molecules with a desired biological activity is an extremely difficult task. In this context, artificial intelligence and generative models have been used for molecular de novo design and compound optimization. Herein, we report a generative model that bridges systems biology and molecular design, conditioning a generative adversarial network with transcriptomic data. By doing so, we can automatically design molecules that have a high probability to induce a desired transcriptomic profile. As long as the gene expression signature of the desired state is provided, this model is able to design active-like molecules for desired targets without any previous target annotation of the training compounds. Molecules designed by this model are more similar to active compounds than the ones identified by similarity of gene expression signatures. Overall, this method represents an alternative approach to bridge chemistry and biology in the long and difficult road of drug discovery.

## Abstract (web summary)

High quality hit identification remains a considerable challenge in de novo drug design. Here, the authors train a generative adversarial network with transcriptome profiles induced by a large set of compounds, enabling it to design molecules that are likely to induce desired expression profiles.

## Introduction

The difficulty of the drug discovery process stems from the fact that only a small fraction of the theoretically possible 10^60^ drug-like molecules are therapeutically relevant^1,2^. One of the most challenging tasks in this scenario is the hit identification, namely the identification of small molecules with an adequate (but usually weak) activity on a specific target that could then be used as a starting point for the chemical optimization process. Hit identification can be achieved by knowledge-based approaches that use previous information coming from endogenous ligands, patents, scientific literature or even structural information of the biomolecule^3^. This task is even more difficult when little or no previous information is available, which usually happens when working with a novel target family or the so called orphan targets. These cases are restricted to serendipity-based (also known as brute-force) methods such as the use of combinatorial libraries or high-throughput screening (HTS)^3^. Although these methods generate copious bioactivity data, they are not very efficient as the amount of resources required is disproportionally large compared to the small number of hits discovered^4^.

One alternative is to use computational methods and data-driven approaches to aid hit identification^4,5^. Techniques such as virtual screening aim to identify hits from virtual libraries containing large number of molecules, usually by similarity-based searches or by molecular docking^4,6,7^. Another technique is automated molecular generation or automated de novo design where new molecules with specific properties are automatically generated by methods such as structure-based de novo design^8,9^, inverse QSAR^10^, particle swarm optimization^11^, or genetic algorithm^12,13^. Recently, artificial intelligence, in particular generative models, has been extensively used for molecular de novo design, compound optimization and hit identification^14–16^. Generative models are very attractive since they can learn the properties of specific real training examples and then automatically generate new synthetic entities with similar characteristics. Several groups in industry and academia have reported the use of recurrent neural networks combined with reinforcement learning as a generative model to design focused compound libraries for HTS with particular physicochemical properties or activity towards a specific target^17–21^. Other generative models such as variational autoencoders (VAE) have been used to automatically optimize molecules to improve their physicochemical and drug-likeness properties^22^. In a similar way, generative adversarial networks (GAN) have been used to produce sets of new molecules with similar properties to known active molecules or photovoltaic materials^23,24^.

Until now, molecular generative models have been designed as chemocentric approaches that barely take into account the resulting biology of the ligand-target interaction. Herein, we report a generative model that bridges systems biology and molecular design. By doing this we can automatically design molecules that have a high probability to induce a desired transcriptomic profile. For this we combine a generative adversarial network with transcriptomic data^25^, which already has been shown to be useful in the identification of new active molecules^26–28^, drug repurposing^29,30^, mode of action deconvolution^31,32^, and prediction of side-effects^33–35^ among other applications. This approach presents several advantages such as the generation of hit-like molecules without the need of previous knowledge of active compounds, biological activity data, or target annotations. In addition, it can be considered as multifunctional since the same model can design molecules for several targets or biological states.

## Results

### Conditioning generative adversarial networks (GANs) with gene expression signatures

GANs are powerful generative models that produce new data points with a similar distribution to that of the real data^36^. These specific networks are composed of two models, namely generator *G*_0_(*z*) and discriminator *D*_0_(*x*), which compete with each other. The generator is optimized to produce new data points similar to those in the real data distribution. In contrast, the discriminator is optimized to distinguish between synthetic data points produced by the generator and those data points coming from the real data distribution. Consequently, at each training step, as the generator tries to produce synthetic data points more similar to the real ones, the discriminator becomes better in distinguishing real data points from synthetic ones.

Due to the great range of applications of GANs, many other extensions to this architecture have been reported. In this work, we used a combination of two of them, namely conditional GANs^37^ and the Wasserstein GAN with gradient penalty (WGAN-GP)^38,39^. In the former one, the generator is conditioned by a variable *c* (*G*_0_(*z,c*)), meaning that the synthetic entities created by the generator will fulfill this condition. In contrast, the WGAN-GP is a variation which minimizes an approximation of the Earth-Mover distance (or Wasserstein-1 distance), instead of minimizing the Jensen–Shannon divergence as in normal GANs. In this particular implementation, the 1-Lipschitz continuity is enforced by using a gradient penalty as an alternative of the gradient clipping scheme (see original paper^39^ for more details). In this way, the final loss functions for *G*_0_(*z,c*) and *D*_0_(*x*) are:

1 $${\mathbf{{\cal{L}}}}_{{\boldsymbol{D}}_0} = \, 	{\Bbb E}_{{\boldsymbol{x}}\sim {\boldsymbol{p}}_{{\mathrm{real}}}}\left[ { - {\boldsymbol{D}}_0\left( {\boldsymbol{x}} \right)} \right] + {\Bbb E}_{{\boldsymbol{z}}\sim {\boldsymbol{p}}_{\boldsymbol{z}},\;{\boldsymbol{c}}\sim {\boldsymbol{p}}_{{\mathrm{real}}}}\left[ {{\boldsymbol{D}}_0\left( {{\boldsymbol{G}}_0\left( {{\boldsymbol{z}},{\boldsymbol{c}}} \right)} \right)} \right]\\ 	 + {\boldsymbol{\lambda }}{\Bbb E}_{{\hat{\boldsymbol{x}}}\sim {\boldsymbol{p}}_{{\hat{\boldsymbol{x}}}}}\left[ {\left( \Vert {\nabla _{{\hat{\boldsymbol{x}}}}{\boldsymbol{D}}_0\left( {{\hat{\boldsymbol{x}}}} \right)_2 - 1\Vert } \right)^2} \right],$$

2 $$\quad\;\; {\mathbf{{\cal{L}}}}_{{\boldsymbol{G}}_0} = {\Bbb E}_{{\boldsymbol{z}}\sim {\boldsymbol{p}}_{\boldsymbol{z}},\;{\boldsymbol{c}}\sim {\boldsymbol{p}}_{{\mathrm{real}}}}\left[ { - {\boldsymbol{D}}_0\left( {{\boldsymbol{G}}_0\left( {{\boldsymbol{z}},{\boldsymbol{c}}} \right)} \right) - {\boldsymbol{\alpha }}{\mathbf{log}}\left( {{\boldsymbol{f}}_0\left( {{\boldsymbol{G}}_0\left( {{\boldsymbol{z}},{\boldsymbol{c}}} \right),{\boldsymbol{c}}} \right)} \right)} \right],$$

where *x* and *c* are a molecule representation and a gene expression signature, respectively, sampled from the real data distribution *p*_real_, *z* is a vector with random noise sampled from a Gaussian distribution (*p*_*z*_) and *f*_0_ is a function (in this case, a neural network) that measures the probability of a gene expression signature corresponding to a molecular representation. The λ and *α* terms are regularization parameters, where the former one balances the influence of the gradient penalty term into the discriminator loss. Similarly, the *α* term weights the influence of the *f*_0_ function in the generator loss. Both, the λ and *α* terms were empirically set to a value of 10.

Recent reports suggest that stacking two or more GANs produce synthetic data with higher definition compared to just using a single GAN^40–42^. In this work we stacked two conditional GANs, where the second one (Stage II) refined the results of the first one (Stage I). The setup of Stage II is similar to Stage I, i.e., it is also composed of a generator (*G*_1_(*s*_0_,*c*)) and a discriminator (*D*_1_(*x*)). The only difference is that instead of taking random noise as input, *G*_1_ takes the output of *G*_0_ (*s*_0_ = *G*_0_(*z*,*c*)) and the gene expression signature (*c*). In this sense, the loss functions for *G*_1_(*s*_0_,*c*) and *D*_1_(*x*) can be written as in Eqs. (3) and (4), respectively.

3 $${\mathbf{{\cal{L}}}}_{{\boldsymbol{D}}_1} = {\Bbb E}_{{\boldsymbol{x}}\sim {\boldsymbol{p}}_{{\mathrm{real}}}}\left[ { - {\boldsymbol{D}}_1\left( {\boldsymbol{x}} \right)} \right] + {\Bbb E}_{{\boldsymbol{s}}_0\sim {\boldsymbol{p}}_{{\boldsymbol{G}}_0},\;{\boldsymbol{c}}\sim {\boldsymbol{p}}_{{\mathrm{real}}}}\left[ {{\boldsymbol{D}}_1\left( {{\boldsymbol{G}}_1\left( {{\boldsymbol{s}}_0,{\boldsymbol{c}}} \right)} \right)} \right]\\ + {\boldsymbol{\lambda }}{\Bbb E}_{{\hat{\boldsymbol{x}}}\sim {\boldsymbol{p}}_{{\hat{\boldsymbol{x}}}}}\left[ {\left( {\left\| {\nabla _{{\hat{\boldsymbol{x}}}}{\boldsymbol{D}}_1\left( {{\hat{\boldsymbol{x}}}} \right)} \right\|_2 - 1} \right)^2} \right],$$

4 $${\mathbf{{\cal{L}}}}_{{\boldsymbol{G}}_1} = {\Bbb E}_{{\boldsymbol{s}}_0\sim {\boldsymbol{p}}_{{\boldsymbol{G}}_0},\;{\boldsymbol{c}}\sim {\boldsymbol{p}}_{{\mathrm{real}}}}\left[ { - {\boldsymbol{D}}_1\left( {{\boldsymbol{G}}_1\left( {{\boldsymbol{s}}_0,{\boldsymbol{c}}} \right)} \right) - {\boldsymbol{\alpha }}\,{\mathbf{log}}\left( {{\boldsymbol{f}}_1\left( {{\boldsymbol{G}}_1\left( {{\boldsymbol{s}}_0,{\boldsymbol{c}}} \right),{\boldsymbol{c}}} \right)} \right)} \right],$$

**Fig. 1:**
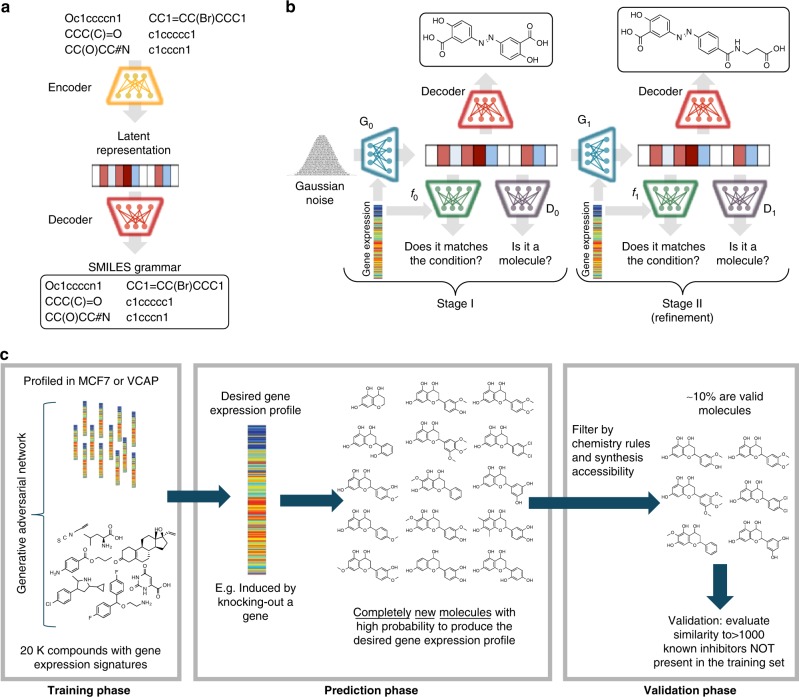
Graphical representation of the models and pipeline used in the study. Molecules were encoded using a model that transforms the canonical SMILES of a molecule into a latent representation that can be later decoded into the set of grammar production rules needed to reconstruct the original SMILES (**a**). The generative adversarial network in **b** has a Stage I where the generator (*G*_0_ in blue) takes the desired gene expression signature together with a vector of random noise to produce a molecular representation that can be decoded into SMILES using the decoder (in red). The discriminator (*D*_0_ in purple) calculates the probability of the molecular representation to be a real molecule and the conditional network (*f*_0_ in green) calculates the probability of the molecular representation to match the gene expression signature. In Stage II, the generator (*G*_1_ in blue) takes as input the desired gene expression signature together with a molecular representation (e.g., the one produced by *G*_0_) to repeat the process. The general pipeline is represented in **c** where the generative adversarial network is trained with ~20 K compounds from the L1000 dataset^25^ (see Methods for details) to be able to generate compounds from a desired gene expression signature during the prediction phase.

All molecular structures were encoded into a vector of continuous values using an approach similar to the one developed by Winter et al.^43^ based on molecular translation. For this we used a SMILES—to—grammar model, which encodes the canonical SMILES representation of a molecule into a latent representation that can be later decoded into the set of grammar production rules needed to reconstruct the original SMILES code (Fig. 1a). This latent representation of the molecule was used to feed real and synthetic molecules into *D*_0_ and *D*_1_, whereas *G*_0_ and *G*_1_ generate this latent representation of synthetic molecules (Fig. 1b).

### Generating molecules from compound-induced gene expression

**Fig. 2:**
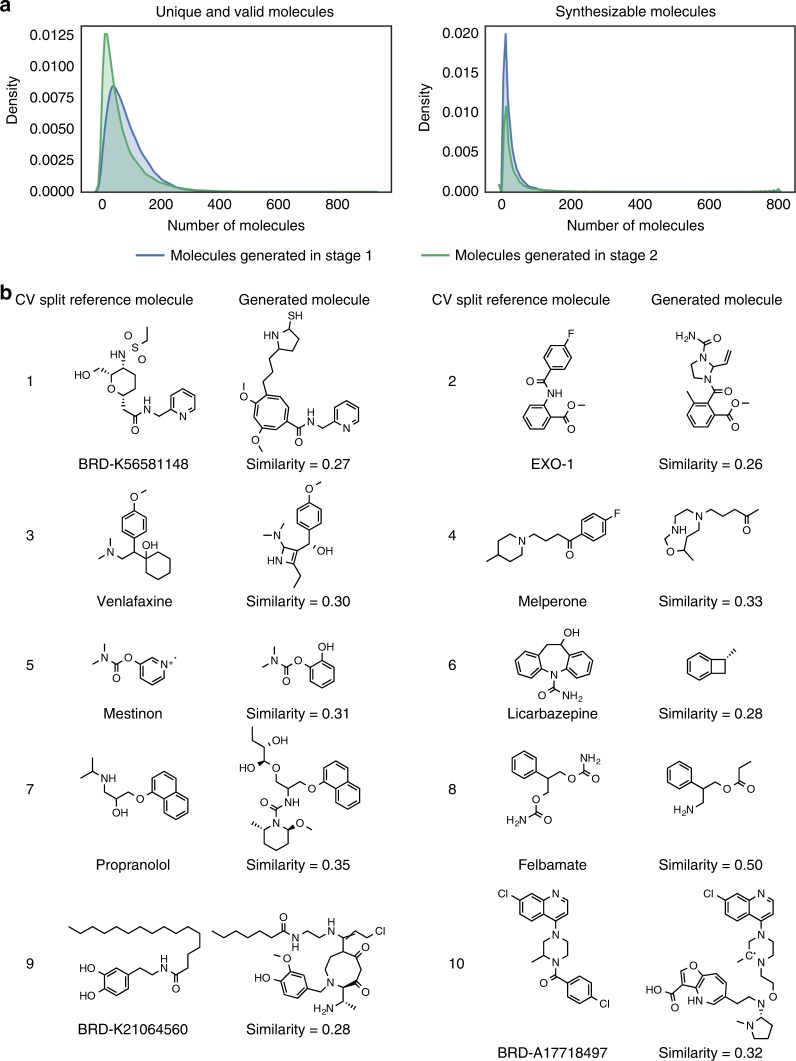
Examples of generated molecules using a compound-induced gene expression signature. **a** Distribution of number of valid and synthesizable molecules generated for each of the 31,821 gene expression signatures used in the 10-fold cross validation scheme. Results of Stage I are shown in blue and for Stage II in green. **b** Examples of generated molecules with their reference compound obtained for each cross-validation split and their respective Tanimoto similarity using Morgan fingerprints.

Molecule generation is challenging, especially when generated molecules are required to meet specific properties. In this case generated molecules were required to induce a particular gene expression signature when exposed to a cell. We evaluated our method with a 10-fold cross validation approach. Specifically, we generated 1000 molecular representations for every ~3000 signatures in each of the validation splits, which were then decoded into SMILES strings. The number of gene expression signatures in the training data (~31,800) is notably larger than the number of compounds (~20,000) as some of these were profiled in more than one condition (i.e., in more than one cell line or concentration). On average, each signature produced ~8.5% of valid molecules, most of them (~8.2% of the total) corresponded to unique SMILES representation, but only a small fraction (~1.6%) were considered easy to synthesize (presenting a synthetic accessibility score^44^ <4.5). Not surprisingly, similar percentages of valid molecules were obtained when sampling points from a latent space using a grammar or character variational autoencoder (7.2% and 0.7%, respectively)^45^. Interestingly, no improvement in the number of generated molecules was observed in stage II compared to the results in stage I of the stacked GAN. Figure 2a shows the distribution of valid and synthesizable compounds generated for each of the 31,821 gene expression signatures used in the 10-fold cross validation. The individual distributions for each cross validation split are also shown in Supplementary Fig. 1.

Following the similarity principle^46,47^ we would have expected that molecules inducing similar gene expression signatures would have, to some extent, similar molecular structures or at least share some pharmacophoric features. Figure 2b shows examples of the generated molecules for each cross validation split and their respective reference compounds i.e., the compound that produced the gene expression signature used as a condition. After measuring the similarity between the reference compounds and their nearest neighbors in the training set (in both molecular and gene expression space) for each cross validation split, we did not find clear evidence that having similar compounds in the training resulted in molecules similar to the reference compound (i.e., the model was not only copying molecules in the training set). Nevertheless, we noticed that the molecular generation, using gene expression profiles of reference compounds with large Euclidean distance to the gene expression profiles used in the training set, usually resulted in molecules with low similarity to the reference compound (Supplementary Fig. 2).

### Designing inhibitor-like molecules using conditioned GANs

**Fig. 3:**
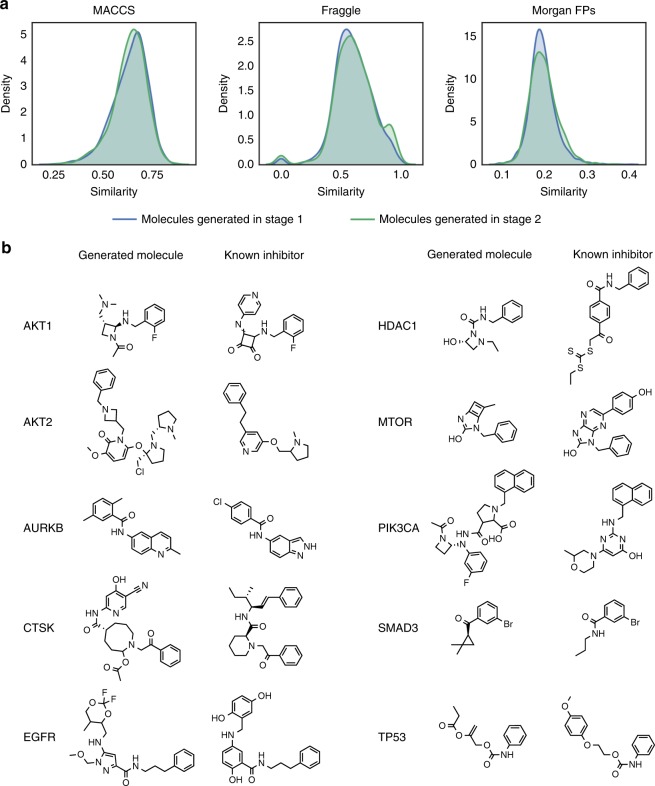
Molecules generated from target knock-out gene expression signatures. **a** Distribution of similarity between all generated molecules and their closest active nearest neighbor using MACCS, Fraggle and Morgan Fingerprints for Stage I in blue and Stage II in green. **b** Chemical structures of some generated molecules and their closest active nearest neighbor for each of the ten different targets.

We evaluated if our approach was capable of generating inhibitor-like molecules only using the gene expression signature of the knocked-out target without any other previous information of the molecular target. The hypothesis being that a knocked-out protein would result in a gene expression signature similar to that observed when the same target protein is inhibited by a potent and selective inhibitor as both situations would, in theory, induce analogous adjustments at the cellular level and therefore equivalent changes in gene expression. Hence the generative model must be able to use the information contained in the knock-out gene signature to generate inhibitor-like molecules. For this, we trained the conditional stacked GAN using all 31,821 compound-induced gene expression profiles and their corresponding compound structures. Then, we generated 1000 molecular representations for each of 148 gene expression signatures induced by the knock-outs of ten protein targets of pharmaceutical interest. As before, there is more than one gene expression signature for each knock-out protein target due to the use of different single guide RNA (sgRNA) for CRISPR knock-outing^25^. All generated molecular representations were filtered to keep only those corresponding to valid and synthesizable molecules. The similarity between the resulting molecules and their nearest neighbor inhibitor contained in the ExCAPE database^48^ was evaluated for each target. Figure 3a shows the distribution of structural similarities between all the generated molecules and their closest known active neighbor not included in the training set. Overall, generated molecules shared similar chemical groups (mean MACCS^49^ similarity = 0.64 ± 0.09) and similar molecular fragments (mean Fraggle similarity = 0.61 ± 0.16) with a known active compound. In fact, the distribution of similarity scores shown by comparing generated molecules to known inhibitors (mean MACCS^49^ similarity = 0.64 ± 0.09) was close to that observed when comparing molecules active on the same targets (mean MACCS^49^ similarity of 0.47 for difficult targets and 0.60 for easy targets) and higher than the one presented when comparing active molecules to random picked compounds (mean MACCS^49^ similarity = 0.4)^50^. It is worth mentioning that 24% of the generated molecules presented a MACCS similarity above 0.7 (but only ~1% above 0.8) to a known inhibitor. Figure 3b shows examples of generated molecules and their closest known active molecules for each of the ten targets. It is surprising to see that in many cases the generated molecule shares functional groups and even a similar molecular scaffold with the active molecule. As seen from these examples, the knock-out gene expression signature of the target was able to direct the molecular generation to specific areas of the chemical space associated with active molecules.

We performed the scaffold analysis to evaluate the potential of the model to generate molecules with known active scaffolds. Only a few scaffolds from the generated molecules (0–14 scaffolds or 5–54 generic scaffolds depending on the target) were also present in active molecules from the ExCAPE database^48^ (Supplementary Table 1). Nevertheless, a high percentage of these (>55% for scaffolds and >65% for generic scaffolds) were not part of the training compounds that are known to be active for these specific targets based on information from the Drug Repurposing Hub^51^. In this sense, the model is doing what it is meant to do: connecting chemistry and biology through gene expression without the need of previous activity labels.

### Optimizing scaffolds towards a gene expression signature

**Fig. 4:**
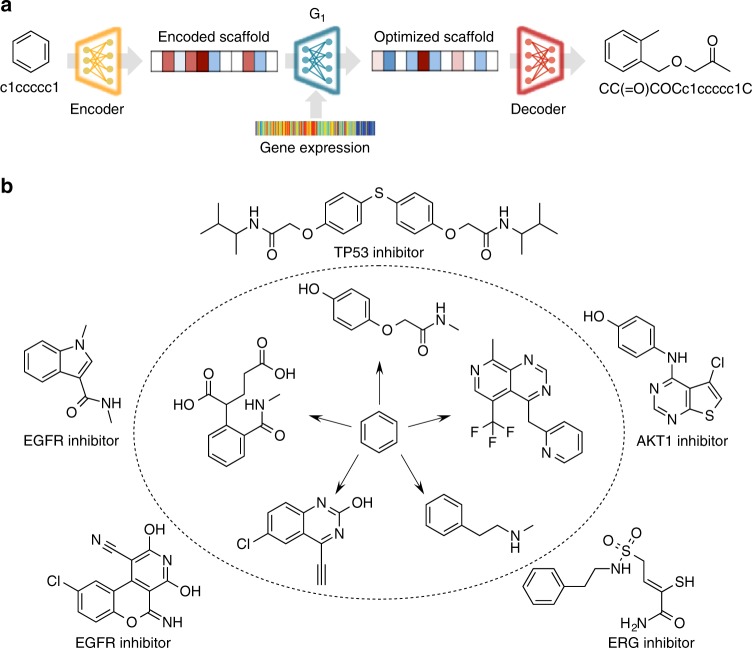
Examples of optimizing the benzene ring scaffold towards different targets using gene expression signatures. **a** The encoder (in yellow) transforms the SMILES of the scaffold into a latent representation that is fed into the Stage II generator (*G*_1_ in blue) together with the desired gene expression signature. The output of *G*_1_ is the latent representation of an optimized molecule that can be decoded into a compound with a high probability to produce the gene expression signature. **b** Molecules generated by optimizing the benzene ring using the knock-out gene expression of AKT1, EGFR, ERG, and TP53 are shown inside the dotted circle and their closest active nearest neighbor outside the circle.

Although that stage II of the stack GAN was designed to refine results from Stage I, it can also be used to refine any other molecule or scaffold. As a proof of concept, we evaluated if the Stage II of our approach was able to optimize the benzene ring (the most common scaffold in the dataset) towards active-like compounds for different targets. For this, we encoded the SMILES of the benzene ring into a latent space representation using the encoder of the SMILES—to—grammar model which then was fed into the Stage II generator (*G*_1_) together with the desired gene expression signature (Fig. 4a). As a result, the model generated an optimized molecule for every scaffold—gene expression signature combination. We repeated this procedure for each of the 148 gene expression signatures corresponding to the ten protein targets previously mentioned. Figure 4b shows some examples of the molecules optimized toward a specific target and their closest active nearest neighbor in the ExCAPE database (not used in the training data). Interestingly, 46% of the resulting molecules kept a benzene ring with the appropriate side chains added by the generative model. Nonetheless, in some cases the generative model also slightly modified the benzene ring producing 11% of the molecules with a pyridine ring. Overall, the generated molecules showed similar molecular fragments to their nearest known active molecule (mean Fraggle similarity = 0.59 ± 0.15), which was a good achievement considering that molecular generation was constrained to a benzene ring as starting point.

### Comparing conditioned GANs with similarity search

Previous studies have performed similarity searches between gene expression signatures induced by different compounds to find molecules that can produce similar effects (e.g., drug repurposing) or between the signatures induced by a compound and a knocked-out target protein in order to find new active molecules^26–28,30,52^. Although there are several success stories using only similarity search, the major constraint of this approach is that the chemical space is restricted to the initial pool of compounds with measured gene expression signatures. In this sense, using a generative model can help to overcome the limitations of the chemical space by generating new compounds tailored to match the query gene expression signature.

**Fig. 5:**
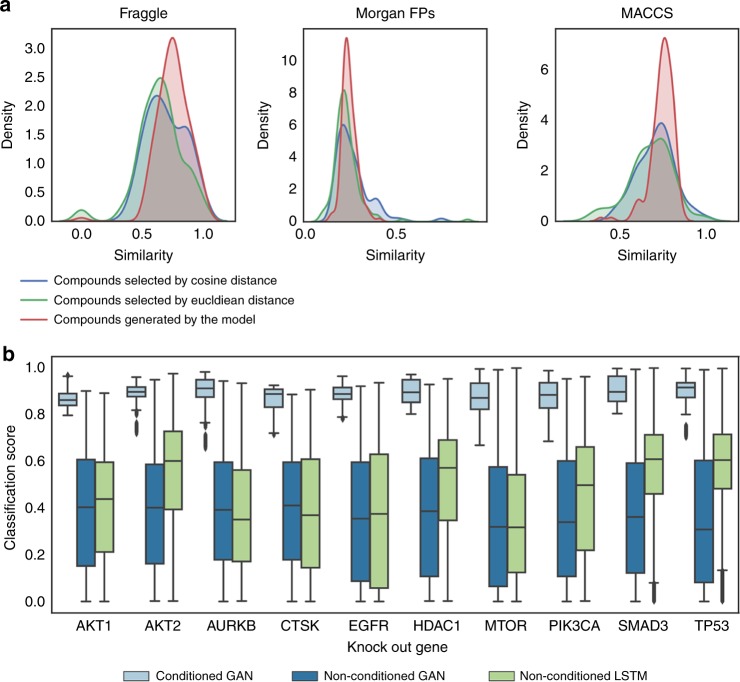
Benchmarking of conditioned generative adversarial network (GAN) with similarity-based search and non-conditioned models. **a** Distribution of structural similarity scores between generated molecules or compounds selected from the training set using similarity search and their closest known active molecule. Conditioned GAN generated more active-like compounds than those found by similarity search using the gene expression signature of a target knock-out. **b** Comparison of conditioned GAN (light blue) with a non-conditioned GAN (blue) and a non-conditioned LSTM (green) to generate compounds for a specific target. The centerline of the boxplot represents the median; the bounds of the box represent the first and third quartile and the whiskers the 1.5 interquartile rage (IQR).

To evaluate the possible advantages of the generative model over the classical similarity search, we compared the ability of these methods to find (or generate) active-like molecules using only the gene expression signature of a target knock-out. For this we used 148 gene expression signatures corresponding to ten target knock-outs. First, we selected the nearest neighbor molecule from the training set by calculating the Euclidean and cosine distances between each knock-out gene expression signature and all compound-induced signatures in the training set. Then, we evaluated the maximum structural similarity between each of the 148 selected molecules and a set of >1000 active molecules for the specific target extracted from the ExCAPE database^48^. At the same time, 1000 molecular representations were produced with the generative model for each of the 148 target knock-out gene expression signatures. After decoding the molecular representations into SMILES and filtering them by validity and synthetic accessibility we chose the most structurally similar generated molecule to one of the >1000 active molecules. Figure 5a shows the distribution of structural similarity between generated molecules or compounds selected from the training set using similarity search (Euclidean or cosine distance) and their closest active molecule in ExCAPE database^48^. The generative model produced molecules which were significantly more similar to active compounds than the ones found by a similarity search using Euclidean distance and the gene expression signature of a target knock-out (*p*-value < 0.001 from a one-sided Mann–Whitney *U*-test for the three different molecular representations). Interestingly, generated molecules also performed significantly better than molecules selected by cosine distance (*p*-value < 0.001 from a one-sided Mann–Whitney *U*-test) when similarity was calculated with MACCS or Fraggle, but not with Morgan fingerprints. Nevertheless, it is important to keep in mind that the better performance of the generative model in these tasks might be due to the fact that similarity search is restricted to the molecules in the training set.

### Conditioned GAN focus on specific areas of the chemical space

An interesting property of the conditioned GAN is that it can guide molecular generation to specific areas of the chemical space that fulfill a specific condition. In this case, generated compounds are conditioned to match a specific gene expression profile and this is measured by the conditional network (*f*_0_) in the form of a classification score i.e., the higher classification score, the better the condition is fulfilled. We used this classification score to compare molecules generated by our conditioned GAN for each of the ten knock-out gene expression signatures (see above) to a set of 450,000 SMILES strings, generated by Segler et al.^17^ using a long short term memory (LSTM) network^53^, that has previously been used as a benchmark^54^. Although the LSTM network was trained on a larger data set (1.4 million compounds from ChEMBL database^55^) and produced a higher number of valid molecules (97.7%), most of them had a low classification score (median value below 0.61) for each of the ten knock-out gene expression signatures (Fig. 5b and Supplementary Table 2). This is not surprising since non-conditioned generative models, like this LSTM network, are trained to produce new data that present a distribution similar to that of the training set. This also explains the wide range of classification scores, where some of them could be higher than 0.8 due to the presence of inhibitors for each of the ten targets in the training set. Similar results were observed when molecules ware generated using a non-conditioned GAN and the L1000 dataset. In contrast, molecular representations obtained from the conditioned GAN showed significantly higher classification scores than the non-conditioned LSTM network for all target knock-outs (*p*-value < 0.001 using a one-sided Mann–Whitney *U*-test). In this case, median classification scores were above 0.85 for all targets. This example shows how conditioning the generative adversarial network can direct the molecular generation process to specific areas of the chemical space that fulfill a condition. It is worth noting that the biological relevance of the targeted region of the chemical space will always depend on the conditional network design and accuracy

## Discussion

In conclusion, we reported a method based on conditional generative adversarial networks that proposes new molecules from a particular gene expression signature. Our method offers some advantages over current molecular generative approaches as well as an alternative way to exploit all the information contained in compound-induced gene expression data, in particular the one in L1000 database. Firstly, this method allows the generation of active-like molecules using just the gene expression signature of the target knock-out. Since no previous measurement of biological activity or target annotations are needed for the training data, this approach could potentially be applied to any target, as we have shown herein. Further work is still needed to assess the optimal biological models to generate the gene expression signatures, especially in the light of the variability of drug responses in cell lines as recently reported^56^. Secondly, we can use the model to modify a chemical scaffold (or other molecule) in order to generate active-like molecules, which is particularly useful for scaffold hopping or compound optimization. Finally, we showed that our method generates molecules more similar to known active compounds than the ones that can be found by a similarity search, which is currently the state-of-the-art method to navigate the L1000 data. The fact that our method does not rely on target annotations or activity data makes it very useful in cases where such information is not available such as in target deorphanization projects. It must be said that there is still room for improve this method, for example evaluating if it can be applied lead optimization or finding ways to generate compounds with known structural features associated with activity on specific drug targets. We expect to apply this method to design directed chemical libraries in order to increase the chances of finding hits in HTS campaigns and therefore, evaluating the performance of this method in a real drug discovery setting. In addition, we are planning to expand this method to automatically generate molecules with multi target signatures or able to reverse toxicological related or disease related gene expression signatures.

## Methods

### Data set

In this study we used the L1000 CMap database recently reported^25^. This database contains induced gene expression profiles of more than 25,200 perturbagens of which ~19,800 are small molecules, 314 biologics and ~5075 genes with altered function by shRNA, cDNA, and/or CRISPR. These perturbagens were assayed in different cell lines to produce around 1.3 million of individual gene expression profiles corresponding to ~473,000 gene expression signatures. Each profile/signature reports the expression of 978 genes (referred as Landmark genes) which can be used to infer the expression of another ~12,000 genes in order to have a better picture of the full transcriptome (see original paper for more details). The complete L1000 CMap data can be downloaded from Gene Expression Omnibus (GEO IDs GSE92742 and GSE70138). In this work we used consensus signatures of Landmark genes coming from perturbagens tested at 5 or 10 µM either on MCF7 or VCAP cell lines after 24 h of exposure. These parameters were chosen in order to maximize the number of data points whilst reducing the dependence on the experimental setup. After applying these filters we ended with 31,821 Landmark gene expression signatures corresponding to 19,768 single compounds, meaning that each compound could have more than one gene expression signature. Finally, the final model was trained on 19,768 compounds and 31,821 gene expression signatures.

### SMILES-to-grammar model

This is a neural machine translation (NMT) model that reads the input SMILES of a molecule (as one-hot encoding), encodes it into a latent representation (vector of continuous values) which can be decoded to the appropriate set of grammar production rules^57^ (http://opensmiles.org/spec/open-smiles-2-grammar.html) so as to reconstruct the original SMILES code. In this case, the encoder is a recurrent neural network (RNN) using a single gated recurrent unit (GRU) cell, the latent representation corresponds to the concatenated cell states of the encoder RNN (256 dimensions), and the decoder is a single GRU followed by a dropout layer (rate of 0.2) and a dense layer (with a softmax activation function), which generates a probability distribution over all possible grammar production rules for each time step. The model was trained following a teacher-forcing^58^ scheme on 1.25 million molecules extracted from ChEMBL 22^55^. It is important to mention that the approach of decoding to grammar production rules was chosen since this can reduce the reconstruction errors when sampling a random molecule as suggested by Kusner et al.^45^

### Generator stage I architecture (*G*_0_(*z*,*c*))

The generator receives as input the condition, in this case a gene expression signature (of 978 genes), and a 1000-dimmensional noise vector sampled from a normal distribution. The two inputs are individually processed by a two-layer multilayer perceptron (MLP) with 512 and 256 nodes, respectively, where each layer uses LeakyRelu as activation function and is followed by a batch normalization procedure. The two resulting tensors are concatenated and used as input for another two-layer MLP, where the first layer has 256 nodes and LeakyRelu activation function. The second layer acts as an output layer (i.e., the number of nodes is equal to the dimensionality of the latent space) and is followed by a tanh activation function.

### Generator stage II architecture (*G*_1_(*s*_0_,*c*))

The generator in stage II of the GAN was designed to refine molecules generated in stage I in two ways, to look more similar to real molecules and to match in a better way the gene expression signature condition. For this reason the architecture of the generator in stage II is based on residual deep networks^59^. This generator receives as input both the gene expression signature (978 genes) and the molecular representation coming from the generator in stage I (*s*_0_). The gene expression data is processed by a two-layer MLP with [512, 256] hidden units, where each layer is followed by a LeakyRelu activation function and batch normalization. The output is then concatenated with the 256-dimensional molecular representation coming from the generator in stage I and fed into a series of residual blocks. In this work, a residual block is defined in the following way:

$$x_{i + 1} = f_i\left( {x_i} \right) + x_i\quad \quad {\mathrm{where}}$$

$$f_i\left( {x_i} \right) = W_2{\mathrm{act}}\left( {W_1x_i + b_1} \right) + b_2$$

where the initial *x*_*i*_ is the concatenation of the molecular representation and the processed gene expression signature, and *W*_1_ and *W*_2_ are trainable weights. The output of the residual block is *x*_*i+*1_, which is used as input of the next residual block. Finally, after a series of residual blocks (*n* = 2 in this work), the output is fed into a dense layer with tanh as activation function, which acts as output layer.

### Discriminator architecture

The discriminator is composed of a four-layer MLP of [256, 256, 256, 1] hidden units with LeakyRelu activation function in the first three layers. In order to reduce overfitting, dropout with rate of 0.4 was used between the second and third hidden layers and between the third and the last layer of the MLP. The same architecture was used for discriminators in both stages (*D*_0_ and *D*_1_).

### Conditional network architecture

The main task of this network was to evaluate the likelihood of a compound (encoded in the latent space) to produce a specific gene expression signature (condition). To this end, the gene expression signature is processed by a MLP of two hidden layers with 512 and 256 units, respectively, and regularized by a dropout layer with rate of 0.4 at the end. In a similar way, the compound latent space coordinates of the compound are also fed into a two-layer MLP with dimension [256, 256] and also finalized with a dropout layer. The outputs of these two MLP, corresponding to the processed gene expression and compound information, were combined using the SubMult+NN comparison function as proposed by Wang and Jiang:^60^

$${\mathrm{Subtraction}} = (m_i - g_i) \odot (m_i - g_i),$$

$${\mathrm{Multiplication}} = m_i \odot g_i,$$

$$h_i = act\left( {W\left[ {{\mathrm{Substraction}},\;{\mathrm{Multiplication}}} \right] + b} \right),$$

where ⨀ operator refers to elementwise multiplication, whereas *m*_*i*_ and *g*_*i*_ are the compound and gene expression information after being processed by their respective MLP. As stated by Wang and Jiang^60^, the subtraction function resembles the calculation of the Euclidean distance before summing across dimensions. In a similar way, the multiplication function is closely related to the cosine similarity but preserving information about independent dimensions. The outputs of these functions are concatenated and used as input of a dense layer followed by an activation function (act, in this work LeakyRelu) to obtain the combined vector *h*_*i*_. Finally, *h*_*i*_ is fed to an output layer that uses a sigmoid activation function that estimates the probability of molecule to produce a certain gene expression signature.

### Training

The conditional generative adversarial network was trained during 1000 epochs using a batch size of 256 (Supplementary Figs. 3–6). Here, an epoch was composed by 125 steps where the weights of the discriminators were updated after each step, whereas those of the generators every ten steps. The network was trained using the RMSprop optimizer with learning rate of 5 x 10^–5^ for both the generator and discriminator in both stages of the GAN. It is also important to mention that stage I and II were trained simultaneously. All neural networks were built and trained using Keras^61^ with a Tensorflow^62^ backend.

### Model evaluation

During training, we generated a molecular representation for each gene signature in the training set at the end of each epoch. These were used to evaluate the similarity between the generated and real molecular representations using Fréchet distance (see Supplementary Fig. 4). The Fréchet distance measures the similarity between two distributions (in this case the real and generated) and was recently proposed as an efficient way to evaluate the efficacy of generative models^54^. This metric takes the mean and covariance of the real distribution (*µ*_r_ and *C*_r_, respectively), together with the mean and covariance of the generated distribution (*µ*_g_ and *C*_g_) in the following formulation:

$$d^2( {( {{\mathrm{\mu }}_{\mathrm{r}},{{C}}_{\mathrm{r}}} ),( {{\mathrm{\mu }}_{\mathrm{g}},{{C}}_{\mathrm{g}}} )}) = \| {{\mathrm{\mu }}_{\mathrm{g}} - {\mathrm{\mu }}_{\mathrm{r}}} \|_2^2 + Tr({{C}}_{\mathrm{g}} + {{C}}_{\mathrm{r}} - 2( {{{C}}_{\mathrm{g}}{{C}}_{\mathrm{r}}} )^{1/2})$$

### Generating molecules from gene expression signatures

As a validation procedure we challenged the model to generate molecules from a gene expression signature coming from a knocked-out target protein. For this, we used 705 gene expression signatures from the L1000 database^25^ corresponding to the knock-outs of 53 target proteins in MCF7 generated by CRISPR technology. Known active molecules for these targets were extracted from the Excape database^48^, where 28 of the 53 protein targets were present and only ten had more than 1000 active molecules. For these ten targets we generated 1000 molecular representation for each gene expression signature (148 in total) and evaluated the model by comparing the generated molecules to the known inhibitors of these targets. We evaluated the similarity between the generated compounds and the known active molecules using Fraggle similarity and Tanimoto similarity using MACCS keys and Morgan Fingerprints (radius = 3, 1024 bits) with RDKIT^63^. It is worth mentioning that during these validation tasks both the number of valid molecules (validity measure) and the number of unique molecules (uniqueness measure) were recorded as sanity check for the generative model.

### Reporting summary

Further information on research design is available in the Nature Research Reporting Summary linked to this article.

## Supplementary information

Supplementary Information

Reporting Summary

## Data Availability

The data that support the findings of this study are available on request from the corresponding author.

## Code availability

The code used to generate results shown in this study is available from the corresponding author upon request.
