# Supplementary material for: De novo generation of hit-like molecules from gene expression signatures using artificial intelligence
Source: Nat Commun. 2020 Jan 3;11:10. doi: 10.1038/s41467-019-13807-w (PMC6941972; doi:10.1038/s41467-019-13807-w)
Supplement: Supplementary file 1 — Supplementary Information [file 41467_2019_13807_MOESM1_ESM.pdf]

De novo generation of hit-like molecules from gene expression signatures using artificial intelligence

Oscar Méndez-Lucio, [a,b] Benoit Baillif, [a] Djork-Arné Clevert, [c] David Rouquié, *,[a] Joerg Wichard *,[d]

[a] Bayer SAS, Bayer Crop Science, 355 rue Dostoïevski, CS 90153 Valbonne, 06906 Sophia Antipolis Cedex, France

[b] Bloomoon, 13 Avenue Albert Einstein, 69100 Villeurbanne, France

[c] Department of Machine Learning Research, Bayer AG, 13353 Berlin, Germany

[d] Department of Genetic Toxicology, Bayer AG, 13353 Berlin, Germany

* These authors jointly supervised this work

E-mail: oscar.mendezlucio.ext@bayer.com, david.rouquie@bayer.com, joerg.wichard@bayer.com

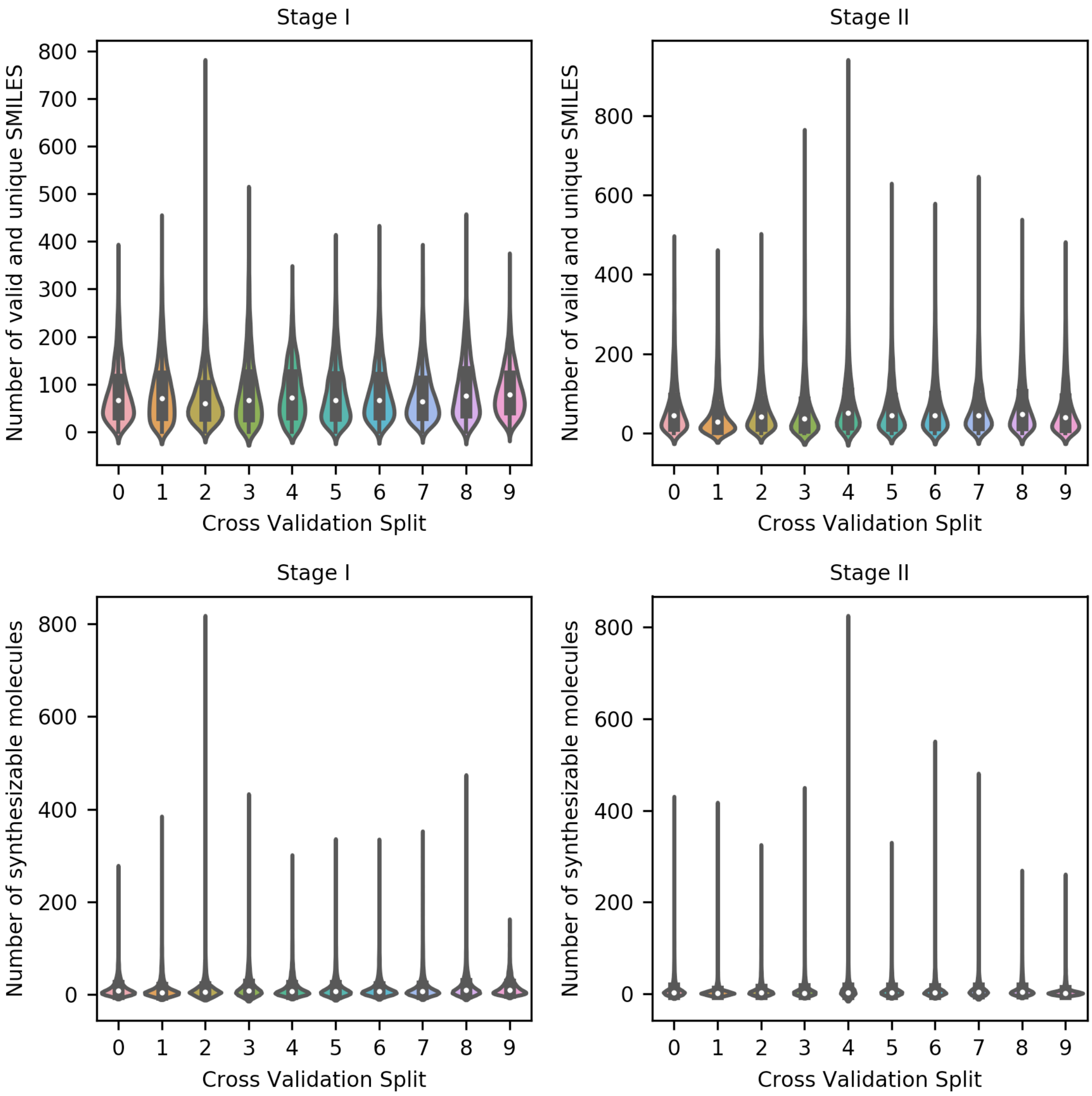

Supplementary Figure 1 Violin plots comparing the distributions of the number of synthesizable molecules and the number valid and unique SMILES for each of the 10 cross validation splits. The median of each distribution is indicated by a white dot.

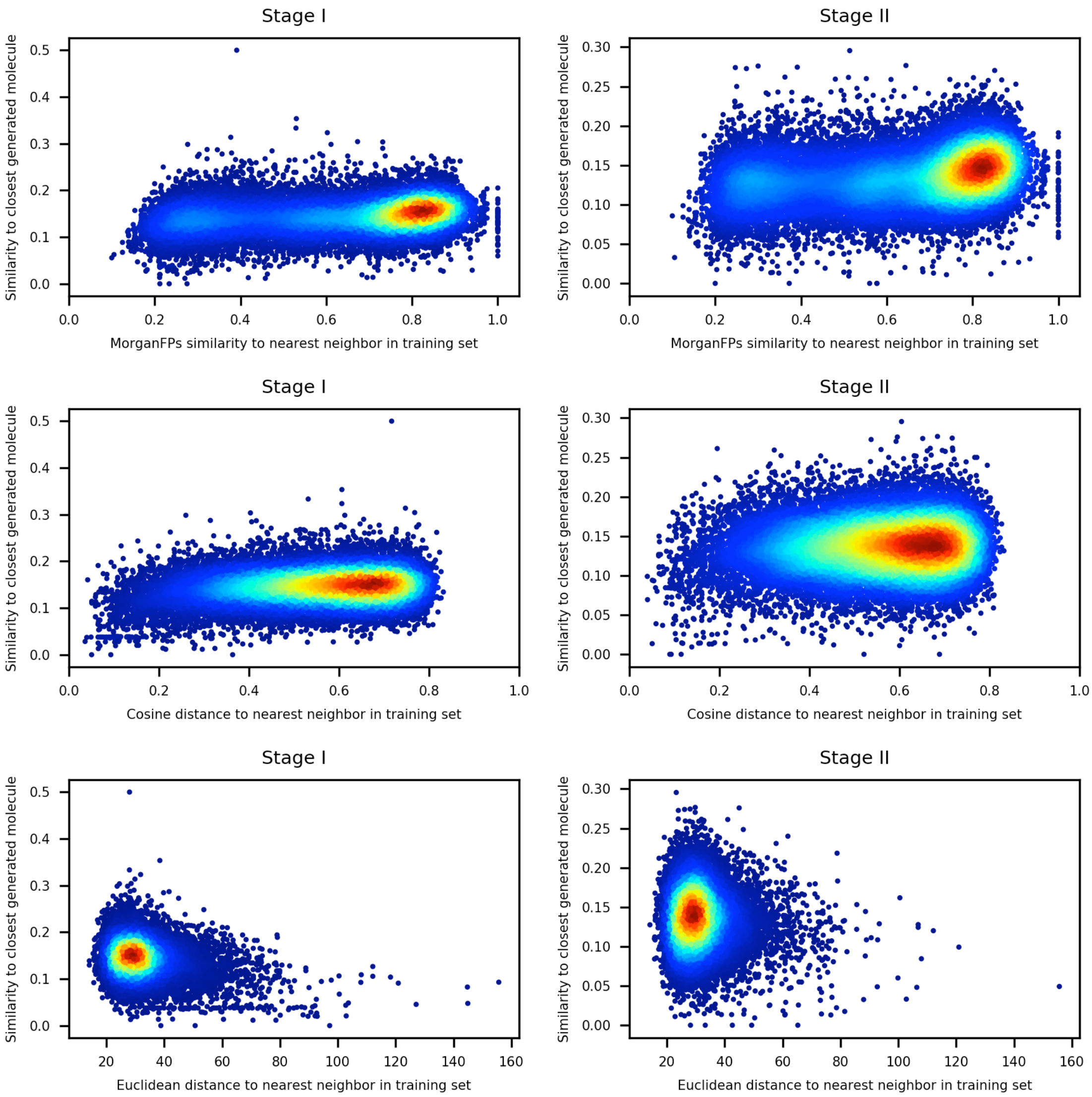

Supplementary Figure 2 Scatter plots showing the similarity between the reference compounds and their nearest neighbors in the training set during cross validation and the similarity between the reference compound and their closest generated molecules (using Morgan FPs). There is not clear evidence that having similar compounds in the training result in similar molecules to the reference compound. Using gene expression profiles of reference compounds with large Euclidean distance to ones the training set usually results in molecules with low similarity to the reference compound. Color represents the normalized density function of the 31,812 data points. Red regions denote densely populated areas whereas dark blue regions sparsely populated areas.

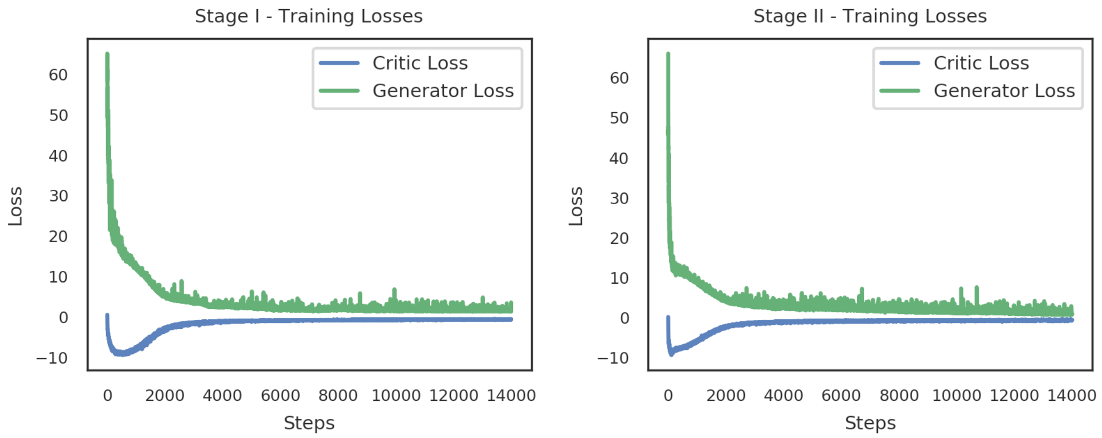

Supplementary Figure 3 Losses of generators and discriminators in Stage I and Stage II for each step in the training process. At the end, discriminator loss is close to zero meaning it cannot differentiate real from generated molecular representations any more.

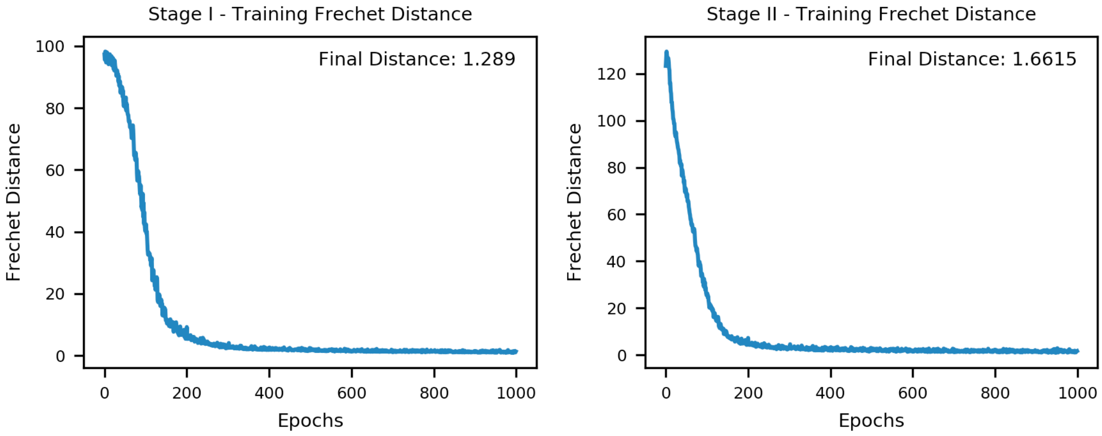

Supplementary Figure 4 Frechet distance between all the real and generated data calculated at each epoch. Distance decreases during training process meaning that at each epoch generators produce molecular representations more similar to the ones of real molecules.

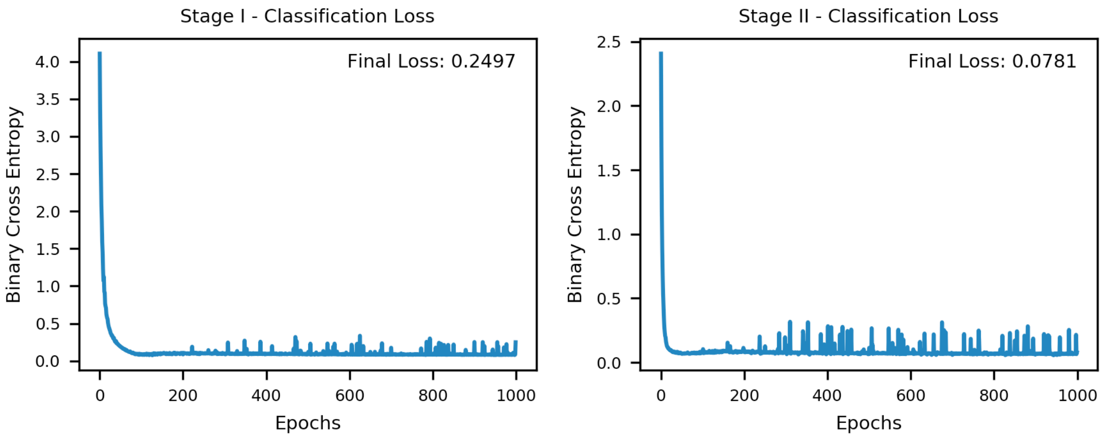

Supplementary Figure 5 Classification loss to evaluate if the generated molecules match or not their conditioning gene expression using the conditional network. Loss decreases very quickly and stay low for all he training losses. This means the generator is fulfilling the condition since very early stages of the training process.

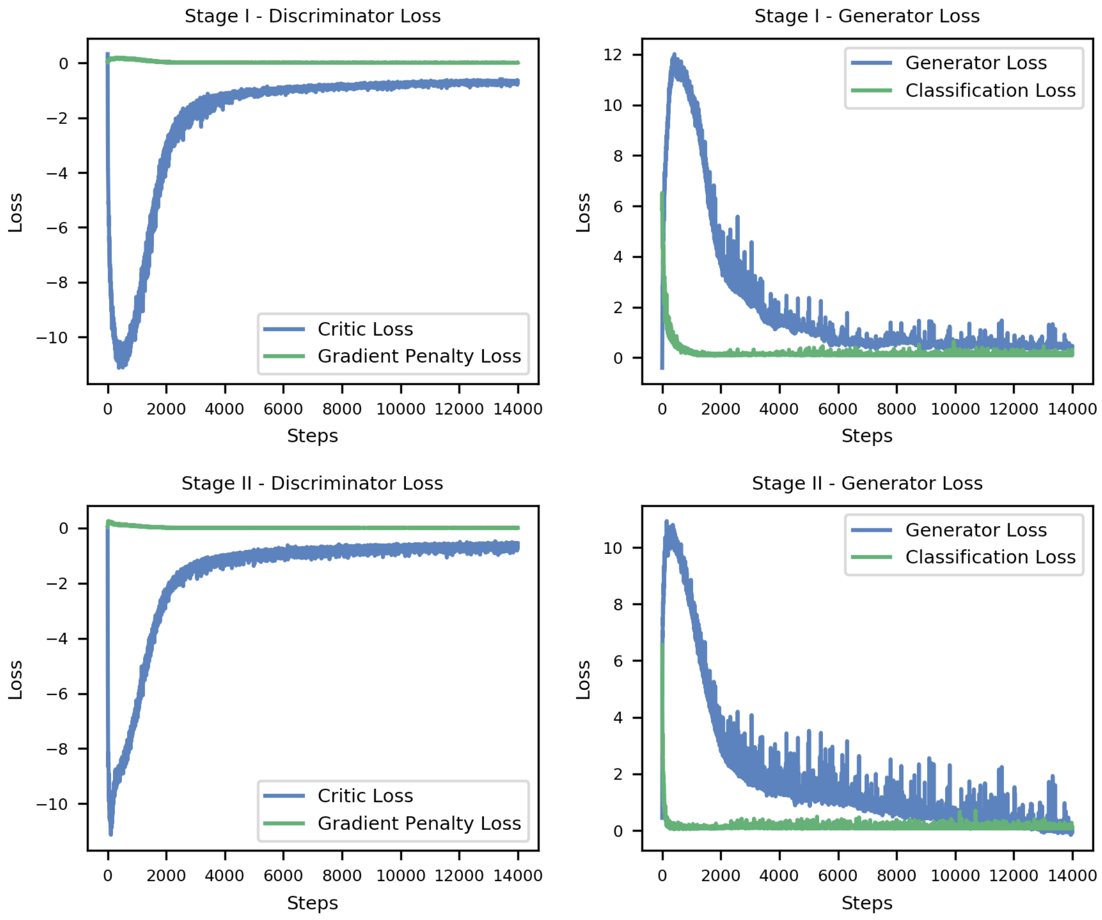

Supplementary Figure 6 Contribution of each term to discriminator and generator loss. The discriminator loss is composed by the critic loss and the gradient penalty. The generator loss is composed by generator loss and the classification loss from the conditioning network.

Supplementary Table 1 Scaffold analysis of generated molecules for each of ten different drug targets. Few scaffolds from the generated molecules were also present in active molecules from the ExCAPE database. High percentage of these were absent of the training compounds that are known to be active for these specific targets based on information from the Drug Repurposing Hub.

Knock-out Gene	Generated scaffolds in a set of known active scaffolds	Generated scaffolds in a set of known active scaffolds but not in known active scaffolds in the training set	Generated scaffolds in known active scaffolds but not in all scaffolds in the training set	Generated generic scaffolds in a set of known generic active scaffolds	Generated generic scaffolds in in a set of known active generic scaffolds but not in known active generic scaffolds in the training set	Generated generic scaffolds in known active generic scaffolds but not in all generic scaffolds in the training set
AKT1	0	0	0	11	8	0
AKT2	0	0	0	6	6	0
AURKB	0	0	0	5	4	1
CTSK	0	0	0	11	9	1
EGFR	4	3	0	18	12	1
HDAC1	7	4	2	25	20	3
MTOR	0	0	0	5	4	0
PIK3CA	0	0	0	7	7	1
SMAD3	14	12	3	49	44	11
TP53	11	8	1	54	46	9

Supplementary Table 2 Median values of classification scores presented by molecules generated with a Conditioned GAN, a Non-conditioned GAN or with a Non-conditioned LSTM. Non-conditioned models showed classification scores < 0.61 whereas conditioned GAN showed significantly higher scores (> 0.85).

Knock-out Gene	Conditioned GAN	Non-conditioned GAN	Non-conditioned LSTM
AKT1	0.857	0.400	0.436
AKT2	0.893	0.399	0.599
AURKB	0.908	0.389	0.349
CTSK	0.883	0.408	0.367
EGFR	0.883	0.352	0.374
HDAC1	0.891	0.383	0.568
MTOR	0.867	0.317	0.315
PIK3CA	0.880	0.338	0.495
SMAD3	0.893	0.359	0.606
TP53	0.912	0.306	0.602
